# Fine particulate matter in acute exacerbation of COPD

**DOI:** 10.3389/fphys.2015.00294

**Published:** 2015-10-23

**Authors:** Lei Ni, Chia-Chen Chuang, Li Zuo

**Affiliations:** ^1^Radiologic Sciences and Respiratory Therapy Division, School of Health and Rehabilitation Sciences, Davis Heart and Lung Research Institute, The Ohio State University College of Medicine, The Ohio State University Wexner Medical CenterColumbus, OH, USA; ^2^Department of Pulmonary Medicine, Ruijin Hospital, School of Medicine, Shanghai Jiao Tong UniversityShanghai, China; ^3^Shanghai Key Laboratory of Meteorology and Health, Pudong Meteorological ServiceShanghai, China; ^4^Interdisciplinary Biophysics Program, The Ohio State UniversityColumbus, OH, USA

**Keywords:** AECOPD, PM_2.5_, oxidative stress, inflammation, alveolar macrophages

## Abstract

Chronic obstructive pulmonary disease (COPD) is a common airway disorder. In particular, acute exacerbations of COPD (AECOPD) can significantly reduce pulmonary function. The majority of AECOPD episodes are attributed to infections, although environmental stress also plays a role. Increasing urbanization and associated air pollution, especially in developing countries, have been shown to contribute to COPD pathogenesis. Elevated levels of particulate matter (PM) in polluted air are strongly correlated with the onset and development of various respiratory diseases. In this review, we have conducted an extensive literature search of recent studies of the role of PM_2.5_ (fine PM) in AECOPD. PM_2.5_ leads to AECOPD via inflammation, oxidative stress (OS), immune dysfunction, and altered airway epithelial structure and microbiome. Reducing PM_2.5_ levels is a viable approach to lower AECOPD incidence, attenuate COPD progression and decrease the associated healthcare burden.

## Introduction

According to the most recent Global Initiative for Chronic Obstructive Lung Disease (GOLD) guidelines, COPD is defined as “a common preventable and treatable disease” characterized by “persistent airflow limitation that is usually progressive and associated with enhanced chronic inflammatory response in the airways and the lung to noxious particles and gases” (GOLD, 2015). Conservative estimates of COPD incidence hover around 210 million cases worldwide, excluding those that are under-recognized or under-diagnosed (Soriano and Lamprecht, 2012). As reported by the World Health Organization (WHO), it is estimated that COPD is poised to become the third largest leading cause of death in the world by 2020 (GOLD, 2015). Due to its high morbidity and mortality, COPD imposes a significant economic burden, contributing to a direct cost of $29.5 billion and an indirect cost of $20.4 billion in the United States annually (National Heart, Lung, and Blood Institute, 2009).

The exacerbation phase of COPD, known as AECOPD, is associated with symptoms such as increased dyspnea, cough, sputum and/or purulent sputum production (GOLD, 2015). AECOPD reduces respiratory function and negatively affects disease progression and prognosis, leading to high healthcare costs (McGuire et al., 2001). Nevertheless, the management of AECOPD remains a clinical challenge. Currently, AECOPD pathogenesis can be attributed to infectious and non-infectious factors. Specifically, bacterial and viral infections are the most prevalent causes of AECOPD (Perotin et al., 2013). In the sputum of 40–60% of AECOPD patients, bacteria such as *Haemophilus influenzae, Moraxella catarrhalis*, and *Streptococcus pneumoniae* can be detected. In addition, airway viral infections, including rhinovirus, respiratory syncytial virus (RSV) and influenza virus may contribute to AECOPD pathogenesis (Perotin et al., 2013).

Non-infectious factors, mainly atmospheric pollution, are also implicated in AECOPD. Notably, inhalable particulate matter (PM), which is a major player in air pollution, is involved in various chronic airway inflammatory diseases. Recent studies have demonstrated that PM_2.5_ (aerodynamic diameter ≤ 2.5 μm) is closely correlated with AECOPD morbidity and mortality (Sarnat et al., 2001; Dominici et al., 2003; Laden et al., 2006). For instance, Li et al. reported that a 10 μg/m^3^ increase in atmospheric PM_2.5_ raised the total mortality of COPD by 2.5% in cities worldwide (Li et al., 2015). PM_2.5_ induces AECOPD through a variety of mechanisms, including oxidative stress (OS), airway inflammation, airway epithelial damage, and inhibition of local airway immunity (Ling and van Eeden, 2009). In this review, we performed a systematic search of PubMed and Medline from 2000 to 2015 for literature related to the role of PM_2.5_ in AECOPD pathogenesis. We aim to investigate PM_2.5_ involvement in AECOPD and the significance of managing air pollution in preventing exacerbations of COPD.

## PM_2.5_ structure and composition

PM refers to the dispersed solid, liquid or solid-liquid suspensions in the air (Wilson et al., 2002). The diameter, composition, and origin of PM play an important role in toxicity and biological pathogenicity (Valavanidis et al., 2008). PM measuring greater than 10 μm in diameter can be trapped in the mucus lining within the nose and respiratory tract, and can be readily eliminated through normal breathing activities (Squadrito et al., 2001). However, PM with a diameter less than 10 μm is more difficult to remove from the respiratory tract when inhaled (Squadrito et al., 2001). In particular, PM_2.5_ (also known as the fine particulate) reaches distal airways and deposits in alveolar regions (Squadrito et al., 2001). The soluble components of PM_2.5_ may enter the blood circulation through alveolar capillaries as ultrafine PM, whereas the insoluble portions sediment in the lung, thereby leading to detrimental health outcomes including airflow obstruction (Ling and van Eeden, 2009; Brook et al., 2010). Consequently, PM_2.5_ is commonly associated with cardiovascular diseases and chronic airway diseases, such as COPD and atherosclerosis. A tendency for PM to accumulate in the centribular emphysematous lesion and induce persistent inflammation has suggested its association with COPD pathogenesis, characterized by emphysema (Ling and van Eeden, 2009). Accordingly, Bose et al. showed the correlations between indoor PM_2.5_ and systemic inflammation in COPD patients. Yet such relationship was not found with coarse fraction of particles (PM_2.5−10_) (Bose et al., 2015).

The extent of PM_2.5_ deposition in the lung is determined by the inhaled concentration, tissue structure, and the clearing ability of airway cilia. COPD patients with persistent airway obstruction and abnormal lung architecture may display higher levels of PM_2.5_ pulmonary deposits when compared with healthy individuals (Ling and van Eeden, 2009). The resulting damage to airway cilia and the reduced ability to perform airway clearance prevent the timely elimination of PM_2.5_ from the airway and lungs (Ling et al., 2011). In a polluted environment, each alveolus comes in contact with an average of 1500 particulate molecules in a 24 h period. Approximately 50% of the PM deposits occur in the alveolus, of which 96% are composed of PM_2.5_ (Valavanidis et al., 2008). Further, the generation of PM_2.5_ is primarily attributed to industrial combustion, traffic emission, and agricultural activities (Laden et al., 2000; Saliba et al., 2010). Its chemical composition varies with different environmental sources.

### PM_2.5_ as a carrier

The characterization of PM_2.5_ chemical composition reveals its toxicity, which is relevant to the adverse health effects imposed by PM_2.5_ (Bell et al., 2007). PM_2.5_ may absorb organic molecules, transition metals, reactive gases, microbial components, and minerals, serving as a carrier for these potentially harmful molecules (Kleeman et al., 2000; Sillanpaa et al., 2006). In particular, Bell et al. have found that several components, including chlorine, zinc, and bromide, showed strong seasonal correlations; that is, higher concentration of these components were detected in PM_2.5_ during winter (Bell et al., 2007).

Iron is a transient metal that is commonly found in PM_2.5_. Soluble metal ions absorbed by PM_2.5_ are considered a key factor in the impairment of lung cell. Redox-active metals trigger a series of catalytic reactions and free radical formation, leading to lipid peroxidation in the cell membrane and inflammation (Harrison and Yin, 2000; Meng and Zhang, 2006; Schwarze et al., 2006). PM_2.5_ containing higher levels of heavy metals may damage the macrophages, leading to reduced phagocytosis (Zhou and Kobzik, 2007). Zhou and Kobzik reported that the chelation of iron substantially reversed its toxicity to macrophage cells (Zhou and Kobzik, 2007). Iron attached to the surface of particulate can disturb redox balance by inducing the release of hydroxyl radicals, further damaging DNA and generating an OS marker 8-hydroxy-2-deoxyguanine (8-OHdG) (Knaapen et al., 2002). High levels of soluble metal zinc in PM have been shown to increase the levels of neutrophils, macrophages and protein in mouse bronchoalveolar lavage (BAL) (Adamson et al., 2000). Adamson et al. also detected focal fibrosis in the alveolar mesenchyme, which demonstrated that zinc in PM_2.5_ was related to chronic or acute pulmonary inflammation (Adamson et al., 2000). Furthermore, Dergham et al. reported that the transient metals, as well as organic chemicals, attached to PM_2.5_ to upregulate the gene expression of xenobiotic-metabolizing enzymes, cytochrome P4501a1 and 1b1 in human bronchial epithelial cells (Dergham et al., 2012).

The biological components of PM_2.5_ are potentially involved in the activation of pulmonary immunity (Alexis et al., 2006). Specially, Alexis et al. observed an up-regulation of TNF-α mRNA expression solely in the non-heat-treated PM_2.5−10_ group (Alexis et al., 2006). During the lipopolysaccharide (LPS)-mediated immune reaction, the expression of mCD14 on the surface of macrophages is higher in the non-heat-treated group than in the heat-treated group (Alexis et al., 2006). It is shown that heat treatment inactivates heat-sensitive biological components on the surface of PM_2.5_ such as LPS (from gram-negative bacteria) and the peptidoglycans (from gram-positive bacteria) (Alexis et al., 2006).

## PM_2.5_ and airway inflammation in COPD

**Figure 1:**
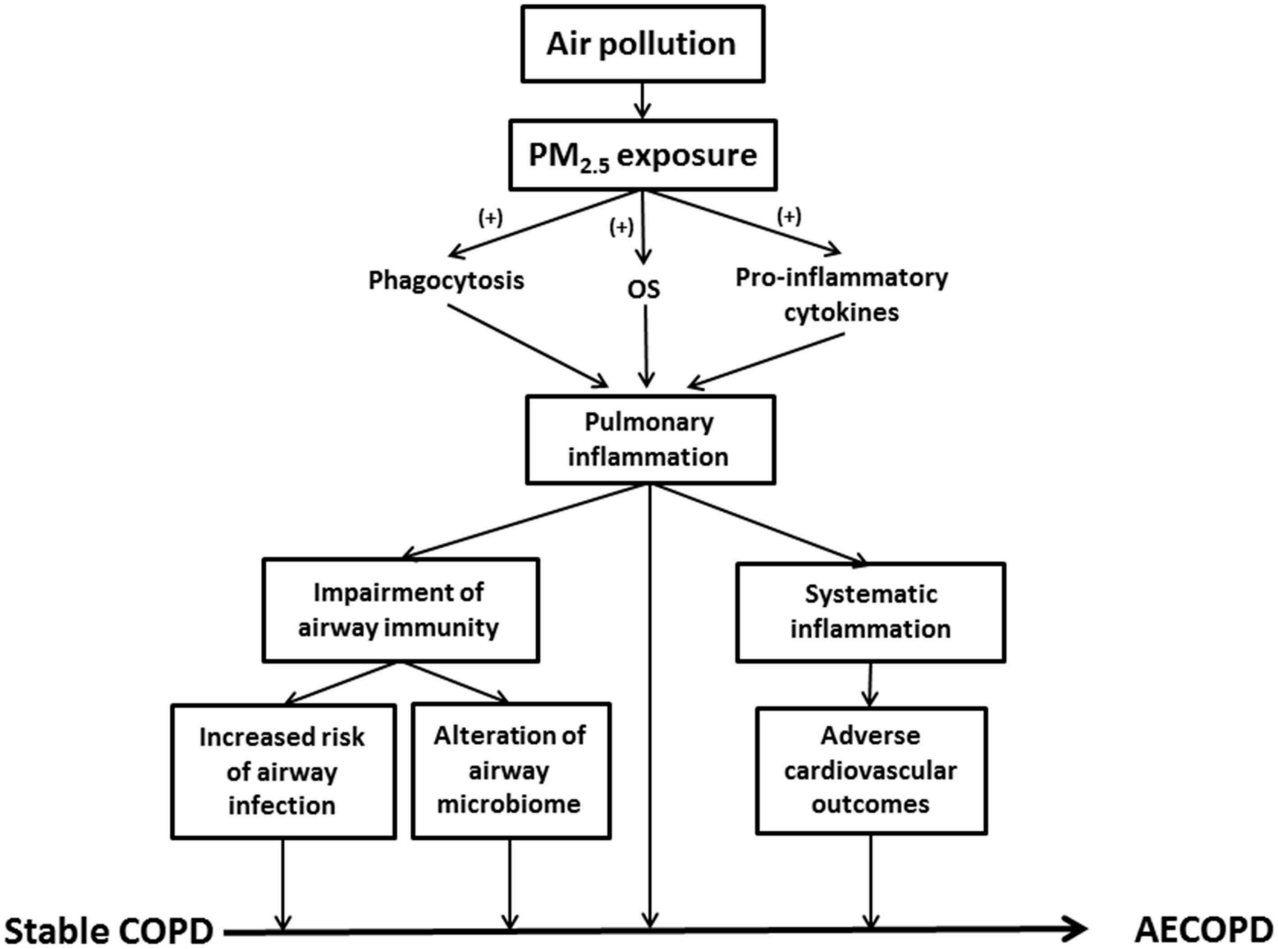
**This schematic summarizes the role of PM_2.5_ in AECOPD**. PM_2.5_, particulate matter ≤ 2.5 μm; OS, oxidative stress; COPD, chronic obstructive pulmonary disease; AECOPD, acute exacerbations of COPD.

Exacerbation of airway inflammation is a key feature in AECOPD (Figure 1). Indeed, inflammatory biomarkers such as tumor necrosis factor alpha (TNF-α), interleukin (IL)-6 and IL-8 were significantly elevated in the induced sputum of AECOPD patients, while the concentrations returned to lower values during the recovery (Bhowmik et al., 2000; Aaron et al., 2001). PM_2.5_ enhanced airway inflammation via any of the following mechanisms (Figure 1): (1) Release of pro-inflammatory cytokines (e.g., TNF-α and IL-1) and oxygen radicals due to active phagocytosis of PM_2.5_ mainly by alveolar macrophages (AMs); (2) Production of chemoattractive and pro-inflammatory mediators, such as granulocyte macrophage colony-stimulating factor (GM-CSF), by lung epithelial cells results in enhanced leukocyte recruitment; (3) Generation of oxygen free radicals and proteases from the damaged alveolar epithelial or immune cells (Ling and van Eeden, 2009; Valavanidis et al., 2013).

Immune cells, such as AMs, directly interact with PM_2.5_, playing an important role in the elimination of inhaled particulate. As mentioned previously, PM_2.5_ may stimulate AM to release inflammatory mediators such as arachidonic acid (AA), TNF-α and IL-6, leading to airway inflammation (Table 1; Pozzi et al., 2003). In addition, PM_2.5_ has been shown to compromise the functional capacity of phagocytic cells and influence the expression of these cells (Ling and van Eeden, 2009). Exhaled nitric oxide (FeNO), an indicator of airway inflammation, is positively correlated with the number of airway inflammatory cells. Notably, the ambient air pollution-induced PM_2.5_ leads to increased FeNO levels in the elderly (Adamkiewicz et al., 2004). In a study with healthy, nonsmoking subjects, an increase in sputum neutrophils was observed after 4 h exposure to diesel exhaust particle (DEP) (Nightingale et al., 2000). Levels of inflammatory factors, including TNF-α, IL-1β, IL-6, and IL-8, were significantly elevated after PM_2.5_ exposure to human bronchial epithelial cells (Cachon et al., 2014).

**Table 1:** **A summary of PM_2.5_ involvement in AECOPD pathogenesis**.

**Potential contributing factors of AECOPD**		**Effects induced by PM_2.5_**	**References**
Inflammation	Airway inflammation	• Neutrophil recruitment to the sputum	Nightingale et al., 2000
		• Stimulate AM to release AA, TNF-α and IL-6	Pozzi et al., 2003
		• NF-κB activation • Elevated inflammatory cytokines	Shukla et al., 2000; Maciejczyk et al., 2010; Cachon et al., 2014
	Systemic inflammation	• Release of white blood cell and platelets	Tan et al., 2000; van Eeden and Hogg, 2002
		• Pro-thrombotic effects • Higher risk of cardiopulmonary events	Mills et al., 2005; Lucking et al., 2008
Oxidative stress		• Airway epithelium-associated OS	Shukla et al., 2000; Baulig et al., 2003
		• Increment of OS metabolites (e.g., 8-isoprostane) and lipid peroxidation (e.g., TBARS) • Reduced GSH and SOD activities	Meng and Zhang, 2006; Riva et al., 2011
		• Redox disruption by transient metals found in PM_2.5_ • Oxidative DNA damage • Reduced phagocytosis due to impaired macrophages	Aust et al., 2002; Knaapen et al., 2002; Zhou and Kobzik, 2007
		• Mitochondrial dysfunction	Upadhyay et al., 2003; Gualtieri et al., 2009; Wu et al., 2013
Altered immunity and susceptibility to infection	Bacterial infection	• Suppressed phagocytosis of bacteria	Lundborg et al., 2006; Zhou and Kobzik, 2007
		• Increased pneumococcal adhesion to epithelial cells	Mushtaq et al., 2011
		• Decreased TLR expressions and impaired antibacterial efficacy	Becker et al., 2005
	Viral infection	• Reduced SP-A and CCSP production	Wang et al., 2003
		• Enhanced viral adhesion and invasion	Castranova et al., 2001; Jaspers et al., 2005

AA, arachidonic acid; AM, alveolar macrophage; CCSP, clara cell secretory protein; GSH, glutathione; IL-6, interleukin 6; NF-κB, nuclear factor kappa-light-chain-enhancer of activated B cells; OS, oxidative stress; SOD, superoxide dismutase; SP-A, surfactant protein A; TBARS, thiobarbituric acid reactive substances; TLR, toll-like receptor; TNF-α, tumor necrosis factor alpha.

PM_2.5_ initiates a series of genetic mechanisms via nuclear factor kappa-light-chain-enhancement of activated B cells (NF-κB) thereby leading to a reaction cascade involving cytokine release (Dagher et al., 2007). PM_2.5_-induced epithelial OS facilitates the binding of NF-κB to DNA resulting in increased mRNA expression of NF-κB-related downstream inflammatory cytokines such as TNF-α, TNF-β, and IL-6 (Shukla et al., 2000). These inflammatory cytokines further increases inflammatory cell infiltration and airway mucus secretion, thereby aggravating airway stenosis and breathing difficulty (Shukla et al., 2000). Additionally, the metals in PM_2.5_ trigger human bronchial epithelial cells to release proinflammatory cytokines via NF-κB activation. PM_2.5_ also induces elevated gene expression of intercellular adhesion molecules, resulting in the adhesion of epithelial and inflammatory cells (Table 1; Maciejczyk et al., 2010).

Systemic inflammation is highly associated with the incidence and progression of COPD (Groenewegen et al., 2008). The levels of blood inflammatory markers, such as c-reactive protein (CRP), fibrinogen, and TNF-α, have been shown to elevate in stable COPD (Gan et al., 2004; Karadag et al., 2008). Whether inhaled PM_2.5_ can directly integrate into systemic circulation still remains a controversy (Brown et al., 2002; Kreyling et al., 2002; Nemmar et al., 2002). PM_2.5_ contributes to the systemic inflammatory response and the functional alteration of multiple organs (van Eeden et al., 2005). PM_2.5_ exacerbates COPD systemic inflammation via: (1) activation of inflammatory cells and related inflammatory mediators that infiltrate into the systemic circulation through lung tissue; (2) stimulation of hepatic synthesis of CRP and fibrinogen; (3) stimulation of bone marrow function leading to increased white blood cell and platelet counts; and (4) damaged endothelial system and increased cardiovascular morbidity and mortality (van Eeden and Hogg, 2002; van Eeden et al., 2005).

### PM_2.5_, airway immunity, and infection

The relationship between PM_2.5_ and airway immune function is complex. PM_2.5_ activates innate immunity and amplifies inflammatory reactions. For example, PM_2.5_ stimulates airway epithelial cells to secrete the chemokine MIP-3α (CCL20), thereby recruiting dendritic cells (DC) to airways (Reibman et al., 2003). In addition, DEP up-regulates the co-stimulatory molecules (CD40 and CD86) and facilitates T helper 2 (Th2)-mediated immune response (van Zijverden et al., 2000). The expression of MHC class II molecule was also up-regulated by DEP exposure (Inoue et al., 2009). Interestingly, DEP exposure prior to viral infection increases the expression of the toll-like receptor 3 (TLR 3) and pro-inflammatory cytokines in human pulmonary epithelial cells, thus enhancing the innate immunity (Ciencewicki et al., 2006). PM_2.5_ exposure alters the distal airway microbiome of COPD patients, and diminishes its ability to resist microbial infection by suppressing the function of immune cells (Figure 1). An elevation of PM_2.5_ level leads to enhanced hospitalization for pneumonia (Tsai and Yang, 2014). According to the study of Matthews et al. PM_2.5_ in urban areas may inhibit the generation of primary T helper type 1 (Th1) cell and reduce the levels of IFN-γ, IL-5, and IL-13, which compromises IFN-γ-mediated antibacterial and antiviral immunity in the lungs (Matthews et al., 2014).

As the first line of host defense in the lungs, AMs are primarily responsible for eliminating harmful microorganisms and inhaled foreign particles, playing an important role in innate immunity (Nicod, 2005). Zhou et al. studied the impact of concentrated ambient particles (CAPs) on murine macrophage cell lines. The results indicated that CAPs suppress the internalization of *S. pneumoniae* by macrophages (Zhou and Kobzik, 2007). In addition, several studies showed that exposure to PM_2.5_ impairs the phagocytic capacity of human AM (Lundborg et al., 2006; Xu et al., 2008). Becker et al. found that PM_2.5_ exposure decreased the expression of phagocytosis-related receptor, CD11b, on the surface of AM, although to a lesser extent compared with PM_10_ (Becker et al., 2003). These studies demonstrated that PM_2.5_ reduced the functional airway defenses when combating viruses, bacteria and other microorganisms by inhibiting the phagocytic function of AM.

Further, PM_2.5_ also affects the function of circulating immune cells. Acute PM exposure stimulates polymorphonuclear leukocytes (PMN)-associated respiratory burst, and superoxide dismutase (SOD) supplementation may diminish such an effect (Marchini et al., 2014). Oxidative DNA damage of the lymphocytes has been reported to significantly associate with transition metal-rich PM_2.5_ (Sørensen et al., 2005). Fujimaki et al. studied the adjuvant impact of DEP on systemic IgE generation in an ovalbumin (OA)-treated mouse model. The experimental group of mice was administered DEP and OA, an antigen that induces allergic pulmonary responses (Fujimaki et al., 2001). After immunization, levels of serum anti-OA IgE antibody were markedly higher in the experimental group than those in the control group treated with OA only. The result indicated that exposure to DEP was responsible for the increased systemic IgE response to OA (Fujimaki et al., 2001). PM_2.5_ is related to airway hyperresponsiveness and systemic IgE response (Ogino et al., 2014). Asthma-COPD overlap syndrome (ACOS), one of the clinical phenotypes of COPD, shows evidence of both airway hyperresponsiveness and fixed airflow obstruction (Zeki et al., 2011). Thus, PM_2.5_ exposure and high levels of serum IgE are risk factors for acute exacerbation of ACOS.

In healthy populations, lower portions of the airways remain aseptic. However, bacterial colonization is commonly observed in the lower airway of stable COPD as well as other chronic lung diseases. Indeed, a higher prevalence of pathogenic infection and altered lung defense have been implicated in exacerbated COPD (Patel et al., 2002; Sethi et al., 2002). Potentially pathogenic bacteria (PPB) present in the BAL fluid include: *S. pneumoniae, Moraxella catarrhalis, Haemophilus influenza*, and *Pseudomonas aeruginosa*. The colonization by PPB is closely related to chronic airway inflammation in COPD (Sethi et al., 2006). PM_2.5_ increases the risk of bacterial infections in COPD patients via several mechanisms. Researchers found that PM_2.5_ particles carry bacteria and bacteria-derived components (Menetrez et al., 2001; Alghamdi et al., 2014). Therefore, they may alter the microbiome in the distal airway of COPD patients and subsequently lead to AECOPD (Figure 1). Another study demonstrated that PM_2.5_ exposure promoted pneumococcal adhesion to human epithelial cells, thereby enhancing pneumococcal pneumonia susceptibility (Table 1; Mushtaq et al., 2011). In addition, suppression of pulmonary immune function is one of the primary factors leading to bacterial infections. The carbonaceous core of DEP absorbs multiple organic materials such as PAH and transient metals. DEP is more likely to be inhaled into lower airways and alveolar tissues (Castranova et al., 2001; Yanagisawa et al., 2003). DEP diminishes pulmonary antimicrobial ability, increasing the susceptibility to infection. PM_2.5_ reduces bacterial elimination from the lungs via suppressed production of LPS-stimulated proinflammatory cytokines, such as IL-1 and TNF-α, and weakened phagocytosis (Castranova et al., 2001). PM_2.5_ decreases the expression of toll-like receptors and suppresses TNF and IL-8 release in AM (Table 1), impairing the antibacterial efficacy of the respiratory system (Becker et al., 2005).

Moreover, *Listeria monocytogenes* (*L. monocytogenes*) is a common gram-positive, facultative anaerobic bacterium. AMs and neutrophils are effector cells that can eliminate *L. monocytogenes* (Yang et al., 2001). Yang et al. observed that DEP reduces the responsiveness of AM to *L. monocytogenes* and its ability to secret antimicrobial oxidants, such as nitric oxide, thus impairing the airway cleaning competence (Yang et al., 2001). Moreover, IL-12 plays an important role in Th1 cell anti-*L. monocytogenes* infection's immune response. Researchers found that DEP decreases cell-mediated immune responses upon *L. monocytogenes* infection via AM-secreted IL-12 suppression (Yin et al., 2002). *S. pneumoniae* infection and sepsis are one of the most common factors for increased hospitalization in the elderly above the age of 70 (Marrie, 2000). *S. pneumoniae* is also a common pathogenic bacterium of AECOPD (Pérez-Trallero et al., 2011). Epidemiological studies have shown that air pollution increases the vulnerability to bacterial pneumonia (Medina-Ramón et al., 2006; Neupane et al., 2010). Zelikoff et al. established rat models through intratracheal instillation of *S. pneumoniae* and observed the impact of PM_2.5_ on the pulmonary antibacterial immune function. The results demonstrated that rats exposed to PM_2.5_ had decreased levels of PMN in BAL fluid (Zelikoff et al., 2003). In addition, the expression of inflammatory mediators such as TNF-α and IL-1 associated with mobilization and activation of PMN, was all decreased (Zelikoff et al., 2003). The mortality of PM_2.5_-exposed group was significantly higher than air-exposed control rats. The study revealed that PM_2.5_ exposure may increase the severity of bacterial infections by inhibiting the host anti-microorganism immunity (Zelikoff et al., 2003).

PM_2.5_ exposure can increase the risk of respiratory viral infections (Wang et al., 2003). Surfactant protein A (SP-A) and clara cell secretory protein (CCSP) are both important components of innate immune defense mediators for antiviral respiratory infection (Wang et al., 2003; Derscheid and Ackermann, 2013). Harrod et al. found that DEP-exposed mice have significantly higher gene expression of RSV in lung tissues than control mice (Wang et al., 2003). In addition, the number of inflammatory cells in BAL fluid of both RSV-infected and uninfected mice that were exposed to high-level DEP, was increased (Wang et al., 2003). Lung histopathology examination revealed airway epithelial remodeling with the loss of the normal appearance of bronchial epithelium, and mucous cell metaplasia. Levels of airway epithelium CCSP production and the SP-A secretion in the DEP group were both reduced (Table 1; Wang et al., 2003). These results suggest that DEP exposure can decrease lung immune defenses against viruses. Likewise, Lambert et al. observed a reduced expression of IFN-γ, a cytokine essential for microbial defenses, in RSV-infected mice preexposed to ultrafine particles, suggesting increased body's susceptibility to RSV upon PM_2.5_ exposure (Lambert et al., 2003). Additionally, DEP exposures can increase the number of infected A549 cells by enhancing OS, the ability of adhesion and invasion of the influenza virus (Jaspers et al., 2005). DEP promotes the replication of the influenza virus in the lungs through the inhibition of antiviral interferon production (Table 1; Castranova et al., 2001). These results support that PM_2.5_ can increase the susceptibility to the influenza virus in airway epithelial cells.

## PM_2.5_, oxidative stress, and AECOPD

The chronic inflammatory reaction and OS in COPD are not limited to the lungs. Instead, systemic oxidant-antioxidant imbalance and low-grade systemic inflammation are observed in COPD (Donaldson et al., 2005; van Eeden et al., 2005). During normal metabolic processes, ROS are formed constantly in the cells (Zuo et al., 2013). Particularly in COPD development, ROS are released excessively by leukocytes, such as macrophages, in the airway due to the enhanced phagocytosis of exogenous molecules. Impaired alveolar epithelial cells also contribute to the elevated level of ROS. Subsequently, the endogenous antioxidants are unable to neutralize overproduced ROS thereby leading to OS (Zuo et al., 2014).

AECOPD patients exhale higher levels of 8-isoprostane in the lungs, indicating pulmonary OS (Biernacki et al., 2003). Several studies have suggested that some components of PM_2.5_, such as transient metals, polycyclic aromatic hydrocarbon (PAH) and carbonaceous material stimulate ROS release from pulmonary epithelial cells. PM_2.5_-induced ROS generation, such as superoxide and hydrogen peroxide, further damage cilia and contribute to mucus hypersecretion, airway stenosis and dyspnea (Dagher et al., 2007; Gualtieri et al., 2010; Seriani et al., 2015). Riva et al. reported that a small dose of PM_2.5_ causes functional and histological lung damage in mouse models. Mice treated with intranasal instillation of PM_2.5_ suspension showed an increased resistance in lung ventilation and a decrease in tissue elasticity (Riva et al., 2011). A pathological examination revealed alveolar collapse and lung biochemical analysis indicated increased neutrophil activity (Riva et al., 2011). Increased OS metabolites, such as 8-isoprostane and thiobarbituric acid, accompanied by elevated catalase activity and a reduction in GSH/GSSG, were also observed (Riva et al., 2011). Accordingly, a dose-dependent decrease of GSH levels and SOD activities was seen in rat lungs exposed to fine particles in a dust storm. The level of thiobarbituric acid reactive substances (TBARS) also increased along with fine particle exposure, indicating the occurrence of lipid peroxidation (Table 1; Meng and Zhang, 2006). Cytokines such as IL-6 and IL-1β induced by ROS/inflammation enter the blood circulation and stimulate liver cells to produce acute phase proteins (e.g., CRP and fibrinogen). As a result, additional systemic inflammation and cardiovascular events are prompted (van Eeden et al., 2005). Meng and Zhang studied the impact of fine particles from a dust storm on systemic OS in a rat model. After 24 h of PM_2.5_ exposure, the antioxidants SOD and GSH were significantly decreased within the lung and liver tissues. TBARS, the biomarkers of endogenous lipid peroxidation, increased in the lungs, heart, and liver demonstrating that PM_2.5_ not only affected the lungs, but also caused systemic OS (Meng and Zhang, 2006).

As the first line of defense, airway epithelial cells are most susceptible to the toxicity of PM_2.5_. Specifically, the damaged epithelium is known to release ROS and inflammatory mediators (Baulig et al., 2003). In an *in vitro* study, which examined the impact of PM_2.5_ on epithelial lung A549 cells, Deng et al. found that these cells release lactate dehydrogenase (LDH), produce ROS, and reduce intracellular levels of SOD and catalase. PM_2.5_-exposed A549 cells also showed an up-regulation of autophagy-related protein markers, such as microtubule-associated protein light chain-3 (LC3), Atg5, and Beclin in both time- and concentration-dependent manner (Deng et al., 2013). These results suggest that PM_2.5_ may damage the lung tissue and impair lung function through epithelial cells autophagy induced by OS. PM_2.5_ significantly induced the mRNA expression of pro-apoptotic genes such as p53 and Bax, while decreasing the mRNA expression of anti-apoptotic genes, such as Bcl-2 in human epithelial lung L132 cells. As a result, PM_2.5_ caused apoptosis of L132 via multiple pathways related to OS (Dagher et al., 2006).

PM_2.5_ may generate a toxic effect in lung epithelium via oxidative damage in the mitochondria (Table 1). Upadhyay et al. reported that airborne PM causes mitochondrial dysfunction in A549 cells via iron-derived free radicals. Reduction in mitochondrial membrane potential and caspase-9 activation was detected in the study (Upadhyay et al., 2003). Alterations in membrane integrity, permeability, and mitochondrial structure result in epithelial cell damage (Gualtieri et al., 2009; Wu et al., 2013). In human epithelial lung cells (L132), PM_2.5_ has been attributed to the elevation of OS markers such as malondialdehyde (MDA) (Garçon et al., 2006). PM_2.5_ also promotes the secretion of TNF-α, inducible nitric oxide synthase (iNOS), and nitric oxide (NO) in a dose- and time-dependent manner (Garçon et al., 2006). Further, PM_2.5_ causes endothelial damage by inducing inflammatory response (Montiel-Davalos et al., 2010). Moreover, Nurkiewicz et al. observed increased LDH activities and albumin protein levels in the experimental rat groups exposing to PM_2.5_ (residual oil fly ash; mean diameter 2.2 μm) (Nurkiewicz et al., 2004).

COPD patients often develop other systemic diseases, including heart disease, degeneration of skeletal muscle function, malnutrition, osteoporosis, diabetes, and depression (Chatila et al., 2008). Cardiovascular diseases such as atherothrombotic disease, pulmonary hypertension, and chronic congestive heart failure, are commonly found in COPD subjects (Sin and Man, 2003; van Eeden et al., 2005). Inflammatory mediators, such as TNF-α, IL-6, IL-1β, and GM-CSF, impair endothelium and elevate white blood cell counts (van Eeden et al., 2005). There were increased levels of PMN as well as leukocyte rolling and adhesion in the microvascular walls (Nurkiewicz et al., 2004). PM_2.5_ exposure may aggravate cardiac afterload by increasing peripheral arterial pressure (Vincent et al., 2001; Nurkiewicz et al., 2004). Epidemiological studies showed that PM_2.5_ is tightly related to cardiovascular incidence and hospitalization (Le Tertre et al., 2002; Dominici et al., 2006). For example, the increased hospital admissions for heart failure is considered to be associated with short-term exposure of PM_2.5_ (Dominici et al., 2006). A multi-country epidemiological study demonstrated that an increase of 10 μg/m^3^ in PM_2.5−10_ size, was associated with a 0.76% increase in cardiovascular deaths and 0.58% in respiratory deaths (Analitis et al., 2006). A study using an animal model showed that short-term exposure to PM_2.5_ causes OS damage in both cardiac and pulmonary tissues (Gurgueira et al., 2002).

Studies have also indicated that systemic inflammation is induced by atmospheric pollution and inhaled PM may stimulate bone marrow to release white blood cells and blood platelets. This can increase blood viscosity, which promotes inflammatory responses and impairments in both pulmonary and cardiovascular tissues (Tan et al., 2000; van Eeden and Hogg, 2002). Since cardiac and pulmonary functions are interrelated, cardiovascular issues may contribute to AECOPD complications. Zuo et al. demonstrated that DEP induces NADPH oxidase-dependent ROS production in a diabetic model of rat cardiomyocyte, thereby exacerbating contractile dysfunction. Such adverse effect can be attenuated by the co-culture with antioxidants (Zuo et al., 2011a,b). Thrombotic events, especially deep vein thrombosis and pulmonary embolisms, are one of the frequent inducing factors and complications of AECOPD (Erelel et al., 2002). There are several ways that PM_2.5_ can alter hemostasis. By utilizing hamster models, Nemmar et al. reported that inhaled DEP can trigger platelet activation and aggregation, promoting thrombus formation (Nemmar et al., 2004). Likewise, the study on human subjects revealed that PM_2.5_ demonstrates pro-thrombotic effects through suppressing tissue plasminogen activator release and impairing endogenous fibrinolysis (Table 1; Mills et al., 2005; Lucking et al., 2008). Therefore, exposure to PM_2.5_ can trigger acute myocardial infarction and atherothrombosis. From the data analysis of Third National Health and Nutrition Examination Survey (NHANES III), researchers found that air pollution is responsible for increased white cell counts, platelet counts, and fibrinogens. These increased inflammatory cells and mediators may potentially lead to higher blood viscosity, further contributing to adverse cardiovascular outcomes (Schwartz, 2001).

## Conclusion

Although the etiology and pathogenesis of AECOPD have not been fully elucidated, studies have shown that air pollution and PM_2.5_ exposure are closely related to AECOPD. PM_2.5_ induces both pulmonary and systemic OS and inflammation, affecting the patient's respiratory and immune function, respiratory microbiome, as well as cardiovascular system. Understanding the mechanisms of PM_2.5_-induced AECOPD contributes to patient risk management, which may reduce the likelihood of acute exacerbations.

### Conflict of interest statement

The authors declare that the research was conducted in the absence of any commercial or financial relationships that could be construed as a potential conflict of interest.

## Abbreviations

COPD: chronic obstructive pulmonary disease
AECOPD: acute exacerbation of COPD
PM: particulate matter
OS: oxidative stress
ROS: reactive oxygen species
AM: alveolar macrophage
DEP: diesel exhaust particle.
